# Modelling the impact and control of an infectious disease in a plant nursery with infected plant material inputs

**DOI:** 10.1016/j.ecolmodel.2016.04.013

**Published:** 2016-08-24

**Authors:** Andrew M. Bate, Glyn Jones, Adam Kleczkowski, Alan MacLeod, Rebecca Naylor, Jon Timmis, Julia Touza, Piran C.L. White

**Affiliations:** a Environment Department, University of York, Heslington, York, UK; b The Food and Environment Research Agency (FERA), Sand Hutton, York, UK; c Department of Computing Science and Mathematics, University of Stirling, Stirling, UK; d Department for Environment, Food and Rural Affairs (DEFRA), Sand Hutton, York, UK; e Department of Electronics, University of York, Heslington, York, UK

**Keywords:** Bioeconomic model, Plant disease, Optimal control, Plant nursery model

## Abstract

The ornamental plant trade has been identified as a key introduction pathway for plant pathogens. Establishing effective biosecurity measures to reduce the risk of plant pathogen outbreaks in the live plant trade is therefore important. Management of invasive pathogens has been identified as a weakest link public good, and thus is reliant on the actions of individual private agents. This paper therefore provides an analysis of the impact of the private agents’ biosecurity decisions on pathogen prevention and control within the plant trade. We model the impact that an infectious disease has on a plant nursery under a constant pressure of potentially infected input plant materials, like seeds and saplings, where the spread of the disease reduces the value of mature plants. We explore six scenarios to understand the influence of three key bioeconomic parameters; the disease's basic reproductive number, the loss in value of a mature plant from acquiring an infection and the cost-effectiveness of restriction. The results characterise the disease dynamics within the nursery and explore the trade-offs and synergies between the optimal level of efforts on restriction strategies (actions to prevent buying infected inputs), and on removal of infected plants in the nursery. For diseases that can be easily controlled, restriction and removal are substitutable strategies. In contrast, for highly infectious diseases, restriction and removal are often found to be complementary, provided that restriction is cost-effective and the optimal level of removal is non-zero.

## Highlights

• The plant trade is an important pathway for the spread of plant pathogens.
• We model a nursery that constantly buys, grows and sells potentially infected plants
• Analytic results give optimal levels of two management tools, restriction and removal.
• For not very infectious diseases, removal and restriction are substitutes.
• For highly infectious diseases, removal and restriction can be complements.

## 1 Introduction

Increases in the movement of people and traded goods as a consequence of globalisation have led to growing concerns about the threat posed by invasive species. especially invasive pathogens of humans, plants and animals (e.g. Anderson et al., 2004, Waage and Mumford, 2008, Perrings et al., 2010, Hulme, 2014, Dalmazzone and Giaccaria, 2014). Recent disease outbreaks in plants, such as the Chalara fungus (*Hymenoscyphus pseudoalbidus*) affecting ash trees across Europe (Pautasso et al., 2013) and the oomycete *Phytophthora ramorum* affecting many plants including larch in Europe (Brasier and Webber, 2010) and oaks in the US (Rizzo et al., 2002), have focused attention on the policy options to reduce the risks of similar plant disease outbreaks occurring in the future, and the management options to reduce damage from newly established pathogen populations. These disease outbreaks have also raised concerns about patterns of plant trade, which has been identified as a key introduction pathway for invasive pathogens (Santini et al., 2013), and on the need for a more prominent role of the private sector in biosecurity practices to mitigate existing risk (Liebhhold et al., 2012). Understanding the economic impacts of damage and mitigation is essential for determining optimal policy and management options for invasive pathogens (Stohlgren and Schnase, 2006).

The body of the literature that combines invasion ecology with economic analysis to deal with these issues has drastically increased in the last decade (for an overview see Olson, 2006, Marbuah et al., 2014). Bioeconomic studies explore the management problem from a central authority perspective, focusing on the potential social welfare benefits from policy intervention to limit the risk of invasive species damages using instruments that include port inspections, quarantine and import tariffs (McAusland and Costello, 2004, Mérel and Carter, 2008), import risk screening programmes (Keller and Springborn, 2014, Springborn et al., 2015), the use of public funds to detect, eradicate and/or control established invaders, and habitat restoration (e.g. Olson and Roy, 2002, Mehta et al., 2007, Sims and Finnoff, 2013). Other studies have examined the trade-off between preventive measures before the arrival and control measures after the invader is known to be in the country in order to determine the optimal allocation of limited public resources between these two strategies (e.g. Leung et al., 2002, Leung et al., 2005, Finnoff et al., 2005, Finnoff et al., 2007, Haight and Polasky, 2010, Sanchirico et al., 2010) Here we add to this literature by adopting a private sector perspective, in order to understand the biosecurity vulnerability and management incentives affecting individual businesses.

One of the challenges for developing policy to reduce the risk of outbreaks of pathogens is the fact that the potential routes of invasion are not only diverse, but also that they are controlled by a mixture of public and private agents. Trading decisions made by private decision-makers may have significant implications for public interest at a regional or national level, but the public costs of an outbreak are likely to far exceed the costs experienced by any one private business, and a privately optimal trading decision is very unlikely to match the publicly optimal one due to potential conflicting interests (Perrings et al., 2005, Mills et al., 2011). Effective control of the risk posed by invasive pest and diseases has been defined as a ‘weakest-link’ public good (e.g. Perrings et al., 2002, Burnett, 2005). Therefore, the risk of outbreak can be in the hands of a single private firm in the trading network. This can limit the level of success of decentralised biosecurity efforts, although it may also allow the firm to take a leadership role, creating incentives for other firms to take action (Hennessy, 2008).

This paper studies the relationship between prevention and control strategies in the context of plant trade. We take a single nursery perspective in order to understand the biosecurity vulnerability and incentives affecting private firms, that can inform subsequent analysis on networks and policy development. We develop a simple bioeconomic model of a private nursery owner who buys in, grows and sells on plants in the face of the threats posed by an infectious pathogen. The management options available to the nursery owner are some combination of (1) restriction, i.e. prevention measures to reduce the number of infected plant materials coming from input sources (for example, inspecting inputs and/or investigating and discriminating input suppliers based on perceived cleanliness) and (2) removal, i.e. taking out infected plants within the nursery. Other means of management like cleanliness and fungicide use are assumed to at constant optimal levels.

Prior bioeconomic research on the plant trade has focused on its role as a significant pathway to the introduction of potentially exotic invasive plants, exploring the use of taxes or annual license fee to reduce this risk and cover the expected environmental damages (Knowler and Barbier, 2005, Barbier et al., 2011). However, implementing these market-based instruments is challenging due to the lack of support among stakeholders in the industry (Barbier et al., 2013, Touza et al., 2014). In this paper, we follow current research on private biosecurity responses to livestock diseases, where disease risk does not only depend on agents’ choices but also is characterised by an underlying epidemiological dynamics (Horan et al., 2010). In this framework, (Horan and Fenichel, 2007) are concerned on the management problem characterised by livestock-wildlife interactions in disease transmission; and (Gramig and Horan, 2011) studied the role of government policies as regular testing on encouraging farmers’ biosecurity investments. More recently, (Horan et al., 2015) focused on assessing whether trade always increase risk or whether it can act as a disease management mechanism.

Our focus, however, is the threat associated with private trading decisions, as infected goods can be bought in and sold on. We contribute to the above work by focusing on plant trade, and addressing the role of both private preventing and controlling behaviour to limit disease transmission risk characterised by epidemiological dynamics. Thus, we examine the potential trade-offs and synergies between these management decisions when the nursery owner's objective is to minimise the expected private costs from infection management and revenue losses associated with the reduced value of infected plants. We find that if the disease spreads faster than the ability to control the disease, removal and restriction complement each other whereas if the disease is controllable, removal and restriction become substitutes.

## 2 Model derivation

### 2.1 Disease dynamics

We consider a plant nursery with a nursery owner who constantly buys plant material, grows it and sells it on when the plant becomes mature (i.e. reaches a target age). A disease is introduced within the input plant material and spreads within the nursery. For simplicity and generality, we assume that the plant population is split into two classes, susceptible plants (*S*) and infected plants (*I*). Infected plants can infect susceptible plants, and once infected a plant remains infected for the rest of its time in the nursery; there is no recovery from the infection.^1^ The consequence of infection for the nursery owner is that infection alters (assumed here to reduce) the net price obtained from selling of a mature plant.

To combat the spread of the infection within the nursery, the nursery owner has two different control measures. The owner can invest (i) in **restriction** to reduce the proportion of infected inputs (be it from inspecting inputs and rejecting infected plants or by selecting suppliers with less infected material); and (ii) in the **removal** of infected plants within the nursery. Removal reduces the time an infected plant stays in the nursery, avoiding additional secondary invasions, but provides no revenue.

**Fig. 1:**
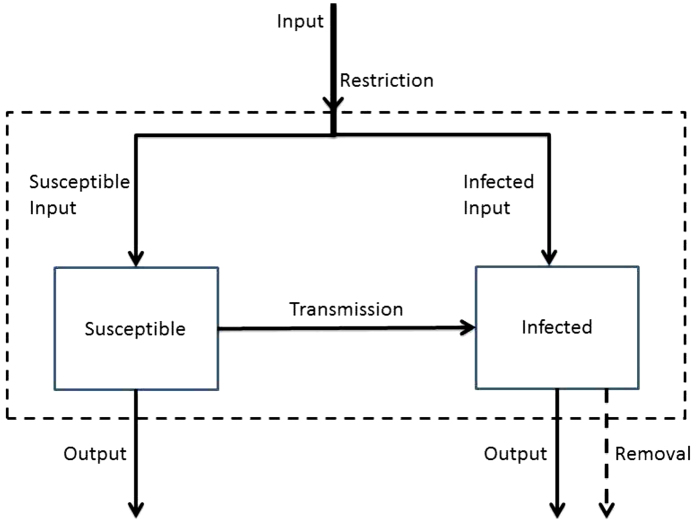
A transfer diagram representing the disease dynamics within the nursery.

Schematically, the plant-disease dynamics can be described as (see Fig. 1):

Change in *S* = Input of *S* − Output of *S* − Disease Transmission,
Change in *I* = Input of *I* − Output of *I* − Removal of *I* + Disease Transmission.

For simplicity, we assume that the stock of plants at the nursery is fixed, *N*, which may mean for example that the nursery is always full (this is a simplifying assumption that is not necessarily realistic; we address this in Section 4). To do this, we set Total Input = Total Output + Removal, where Output of *S* = *δS* and Output of *I* = *δI*, where *δ* is the rate of plants become mature and sold off (i.e. plants stay for an expected time of *δ*^−1^ in the absence of removal).^2^ This means instantaneous replacement of any removed plant is assumed; when something is either sold or removed by control, it is immediately replaced to keep the stock at nursery constant. We also set removal as proportional to the infected plant stock, i.e. removal of *I* = *u*_*rem*_*I*, where *u*_*rem*_ is removal control effort (with units of removal effort per infected plant per unit time). We will assume that *u*_*rem*_ is bounded between 0 and *u*_*remmax*_, the maximum possible effort spent on removal. Incorporating this, we have:

(1) $$\mathrm{Total}\;\mathrm{Input}=\delta (S+I)+u_{\mathrm{rem}}I.$$

This input is split between susceptible and infected plants; *p*(*u*_*ins*_) is the proportion of plant inputs that are infected (as a function of restriction effort per unit time *u*_*ins*_, which is a control variable) and thus (1 − *p*(*u*_*ins*_)) is the proportion of plant inputs that are susceptible.

Incorporating the control measures into standard SI equations (Kermack and McKendrick, 1927, Anderson and May, 1991, Britton, 2003), and assuming density dependent transmission (*βSI*), we get:

(2) $$\frac{\mathrm{dS}}{\mathrm{dt}}=(1-p(u_{\mathrm{ins}}))(\delta (S+I)+u_{\mathrm{rem}}I)-\delta S-\beta \mathrm{SI},$$

(3) $$\frac{\mathrm{dI}}{\mathrm{dt}}=p(u_{\mathrm{ins}})(\delta (S+I)+u_{\mathrm{rem}}I)-\delta I-u_{\mathrm{rem}}I+\beta \mathrm{SI}.$$

Given the assumption of constant total plant stock at the nursery (*S* + *I* = *N*), we can reduce the system down to one equation by substitution *S* = *N* − *I*. We can also rescale the infected population by the total population and consider disease prevalence, *i* = *I*/*N*, the proportion of infected plants in the population (0 ≤ *i* ≤ 1).

Then we get:

(4) $$\frac{\mathrm{di}}{\mathrm{dt}}=\frac{1}{N}\frac{\mathrm{dI}}{\mathrm{dt}}=p(u_{\mathrm{ins}})(\delta +u_{\mathrm{rem}}i)-\delta i-u_{\mathrm{rem}}i+\beta N(1-i)i.$$

Furthermore, we rescale time by *δ*^−1^, the expected time a susceptible plant stays in the nursery. Consequently, *τ*(=*δt*) is the number of generations. Thus:

(5) $$\frac{\mathrm{di}}{d\tau }=p(u_{\mathrm{ins}})(1+{\hat{u}}_{\mathrm{rem}}i)-i-{\hat{u}}_{\mathrm{rem}}i+R_{0}(1-i)i,$$

where ${\hat{u}}_{\mathrm{rem}}=u_{\mathrm{rem}}\delta^{-1}$, the removal effort per plant generation (which is bounded above by ${\hat{u}}_{\mathrm{remmax}}=u_{\mathrm{remmax}}\delta^{-1}$), and *R*_0_ = *βNδ*^−1^, the basic reproductive number, the expected number of secondary infections from a single infected plant over the lifespan of the infected plant in the nursery in an otherwise wholly susceptible plant stock. The basic reproductive number is fundamental to whether a disease will spread and is discussed in Section 3.

As mentioned previously, the proportion of plants brought into the nursery being infected (*p*(*u*_*ins*_)) is a function of restriction (*u*_*ins*_). We assume that the proportion of infected plant inputs has the following properties:

• *p*(*u*_*ins*_) is a continuously differentiable function of the restriction effort *u*_*ins*_.
• With no restriction of plant inputs (*u*_*ins*_ = 0), some proportion of infected plants, *a*, will enter the nursery, i.e. *p*(0) = *a* where *a* ∈ (0, 1].
• With any finite restriction effort, some proportion of infected plant will enter the nursery, i.e. *p*(*u*_*ins*_) > 0 for all finite *u*_*ins*_. This means that it is not possible to completely stop infected inputs from arriving no matter how high the level of effort, be it from the difficulty to recognise asypmtomatic infected inputs, or machine and human error.
• For all restriction efforts, increasing restriction effort reduces the proportion of infected plant entering the nursery, i.e. *p*(*u*_*ins*_) is a monotonically decreasing function of *u*_*ins*_ (equivalently, $\frac{\mathrm{dp}}{du_{\mathrm{ins}}}\leq 0$ everywhere).

**Fig. 2:**
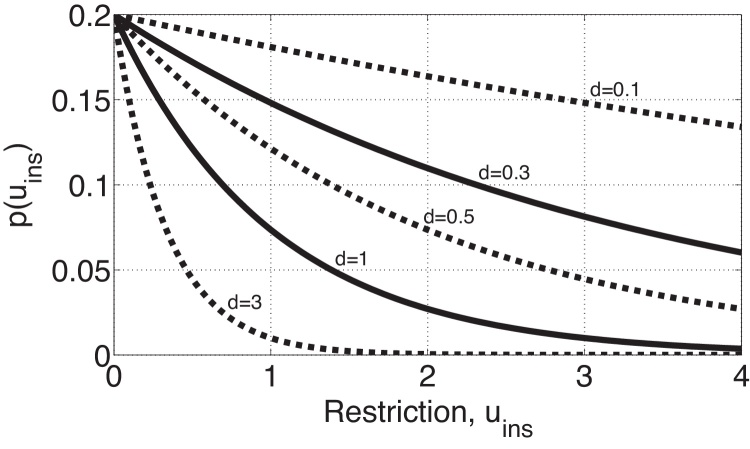
Proportion of infected plant inputs, *p*(*u*_*ins*_), where *p*(*u*_*ins*_) = (*a* − *b*)*exp*(−*du*_*ins*_) + *b* with *a* = 0.2, *b* = 0 and various of values of *d*. The solid lines are values used in Scenarios found in Section 3.

Any function that is (a) continuous, (b) bounded below (by zero in this case) and (c) monotonically decreasing, must converge to some limit as *u*_*ins*_ goes to infinity. We denote this limit *b*, the proportion of inputs that are infected when unlimited restriction effort is used, where *b* ∈ [0, *a*]. A simple candidate that satisfies all of these characteristics is *p*(*u*_*ins*_) = (*a* − *b*) exp(−*du*_*ins*_) + *b*, is plotted in Fig. 2 for various values of *d*, where *d* can be interpreted as the effort-effectiveness of the restriction measures, i.e. the reduction in the proportion of infected plant inputs per unit of restriction effort.

### 2.2 Bioeconomic model

We consider a price-taking representative nursery owner who seeks to maximise profit, faced with the impact of an infectious plant disease. In our model, two types of outputs are taken into account: fully matured susceptible and infected plants with *P*_*S*_ and *P*_*I*_ representing the unit net price of those outputs, respectively.^3^ We assume that *P*_*I*_ < *P*_*S*_ since the infection would likely decrease the plants value when mature and could incur higher production costs.^4^ The dynamics of the proportion of infected plants within the nursery is given by Eq. (5). In addition, we assume that disease symptoms become more apparent as infected plants mature. This, together with an assumption of a regime of inspections within the nursery (inspection regime is independent of the state of the nursery, i.e. a constant cost and thus can be ignored), leads to the nursery owner having good knowledge of which plants are infected and so can act accordingly if desired. All the mature plants sold, or those subject to removal control, are immediately replaced given a constant price *P*_*in*_ of plant inputs. This is consistent with our earlier assumption of constant stock within the nursery.

We also consider the costs of removing infected plants and undertaking restrictions measures to prevent buying infected input plant material. The cost of removing infected plants should increase both with the number of infected plants and with the removing control effort, *u*_*rem*_. Consequently, we will assume for simplicity that the cost of removing infected plant is linearly dependent on the number of infected plants and to prevent the unfeasible case of unbounded removal control effort, we will set a maximal value of removal control effort of *u*_*remmax*_. Similarly, the cost of the restriction regime is proportional to the restriction effort *u*_*ins*_, assumed to be dominated by fixed costs and thus is independent from the level of removal effort and number of infected plants (i.e. there is no additional cost from restricting measures when buying input material to replace the removed infected plants).

The management decision problem is to maximise the present value profits by selecting the level of control in restriction and removal measures over the time horizon *T* and is characterised by the optimising equation:

(6) $$\max_{u_{\mathrm{ins}},u_{\mathrm{rem}}}\mathrm{Profit}=\int_{0}^{T}\overset{\text{Discounting}}{\overset{︷}{e^{-\mathrm{rt}}}}(\overset{\text{Revenue from selling S}}{\overset{︷}{P_{S}\delta S}}+\overset{\text{Revenue from selling I}}{\overset{︷}{P_{I}\delta I}}-\overset{\text{Purchase of replacement stock}}{\overset{︷}{P_{\mathrm{in}}(\delta N+u_{\mathrm{rem}}I)}}-\overset{\text{Cost of removing}}{\overset{︷}{c_{\mathrm{rem}}u_{\mathrm{rem}}I}}-\overset{\text{Cost of restriction}}{\overset{︷}{c_{\mathrm{ins}}u_{\mathrm{ins}}}})\mathrm{dt}$$

subject to Eqs. (2), (3) where *u*_*rem*_ ∈ [0, *u*_*remmax*_] and *u*_*ins*_ ≥ 0, and where *r* is the discount rate. Eq. (6) is very amenable to analytic techniques around static solutions if we focus on the terms within the brackets. This means ignoring the discounting terms and the effects around terminal and initial conditions by assuming the terminal time is large enough for dynamics solution to have converged to the static solution. These static solutions will be the focus of this paper. Appendix C demonstrates that taking the Hamiltonian approach with optimal conditions used in much of the economic literature (using Pontryagin's maximum principle Pontryagin, 1987) and then assume constant controls, will arrive at the same optimality conditions, perturbed by a term proportional to the discount rate (which is rescaled to $\hat{r}=\frac{r}{\delta }$). This discounting perturbation should be negligibly small since plant nurseries usually keep plants for a few months, possibly up to a couple of years.

Taking the static problem, and rescaling parameters and variables in (6) as for (5), we get:

(7) $$\max_{{\hat{u}}_{\mathrm{ins}},{\hat{u}}_{\mathrm{rem}}}P_{S}(1-i)+P_{I}i-P_{\mathrm{in}}(1+{\hat{u}}_{\mathrm{rem}}i)-c_{\mathrm{rem}}{\hat{u}}_{\mathrm{rem}}i-{\hat{u}}_{\mathrm{ins}}$$

subject to Eq. (5) where ${\hat{u}}_{\mathrm{rem}}=u_{\mathrm{rem}}\delta^{-1}\in [0,{\hat{u}}_{\mathrm{remmax}}]$ (as before), ${\hat{u}}_{\mathrm{remmax}}=u_{\mathrm{remmax}}\delta^{-1}$ and ${\hat{u}}_{\mathrm{ins}}=c_{\mathrm{ins}}u_{\mathrm{ins}}{\left(\delta N\right)}^{-1}$**).**

Note that *u*_*ins*_ has been rescaled to ${\hat{u}}_{\mathrm{ins}}$, which now represents restriction control costs (with units of restriction cost per plant in nursery per unit time). Thus, we need to define the proportion of infected inputs as a function of this rescaled restriction control cost. For the case *p*(*u*_*ins*_) = (*a* − *b*) exp(−*du*_*ins*_) + *b*, as $\hat{p}({\hat{u}}_{\mathrm{ins}})=(a-b)\exp(-\hat{d}{\hat{u}}_{\mathrm{ins}})+b$ where $\hat{d}=d\delta Nc_{\mathrm{ins}}^{-1}$ such that $\hat{p}({\hat{u}}_{\mathrm{ins}})=p(u_{\mathrm{ins}})$. Here $\hat{d}$ represents the cost-effectiveness of restriction efforts, i.e. the reduction in the proportion of infected inputs per dollar invested in restricting measures.

Given some terms are constant and thus have no influence on the optimised solution, we can simplify slightly and gather terms in the objective function (7) to arrive at

(8) $$\max_{{\hat{u}}_{\mathrm{ins}},{\hat{u}}_{\mathrm{rem}}}\left(\overset{\text{revenue lost from infecteds}}{\overset{︷}{P_{I}-P_{S}}}-\overset{\text{costs of removing and replacing infecteds}}{\overset{︷}{(P_{\mathrm{in}}+c_{\mathrm{rem}}){\hat{u}}_{\mathrm{rem}}}}\right)i-\overset{\text{restriction costs}}{\overset{︷}{{\hat{u}}_{\mathrm{ins}}.}}$$

Eq. (8) can be simplified further by setting *L* : = *P*_*S*_ − *P*_*I*_ and *C* : = *P*_*in*_ + *c*_*rem*_. Therefore, *L* is the loss incurred from selling a mature infected plant instead of a mature susceptible plant, whereas *C* is the total cost of removing which includes both the expenses associated with the removal and replacement of an infected plant. Using this notation, it becomes clear that the nursery owner management problem consists of minimising the loss in revenue due to selling infected plants and the costs of management (removal and restriction). To simplify notation further, we will henceforth remove all the hats (i.e. set ${\hat{u}}_{\mathrm{rem}}$ as *u*_*rem*_, ${\hat{u}}_{\mathrm{ins}}$ as *u*_*ins*_, $\hat{p}({\hat{u}}_{\mathrm{ins}})$ as *p*(*u*_*ins*_) and $\hat{d}$ as *d*). Consequently, the nursery management decision is to choose between the two control strategies to minimise these costs of the infection,

(9) $$\min_{u_{\mathrm{ins}},u_{\mathrm{rem}}}Q:=(L+{\mathrm{Cu}}_{\mathrm{rem}})i+u_{\mathrm{ins}}$$

subject to

(10) $$\frac{\mathrm{di}}{d\tau }=p(u_{\mathrm{ins}})(1+u_{\mathrm{rem}}i)-i-u_{\mathrm{rem}}i+R_{0}(1-i)i,$$

where *u*_*ins*_ ≥ 0 and *u*_*rem*_ ∈ [0, *u*_*remmax*_].

### 2.3 Analysis

We start the analysis of the system (9), (10) by looking at the long term disease dynamics for a given constant control regime. We compare the case where restriction is perfect, i.e. all plant inputs are susceptible (*p*(*u*_*ins*_) = 0) with a case where restriction is imperfect, i.e. some plant inputs are infected (*p*(*u*_*ins*_) > 0). Following this, we derive the necessary conditions describing optimal level of effort in restriction and removal strategies, using the equilibrium found in Section 3.1.2. Subsequently, we demonstrate some of the theoretical results with numerical solutions. For simplicity, we will focus on exploring how the optimal level of management changes with respect to changes in key parameters: the basic reproductive number (*R*_0_), the loss in revenue from selling an infected mature plant (*L*) and the cost-effectiveness (*d*) (the decay in the proportion of infected plant inputs per dollar spent in restriction efforts) and keep all other parameters fixed. This means, as a baseline, we assume that (i) the background level of infection within the input plant material is *a* = 0.2, so the disease is widespread within the traded plant material; (ii) it is possible to restrict all infected inputs with unlimited restriction *b* = 0, and (iii) the cost of removing and replacing an infected is set at *C* = 10. The nursery's maximum level of effort on removal is assumed to take any value up to *u*_*remmax*_ = 6.

For the basic reproductive number, we will consider two cases, *R*_0_ = 0.5 (i.e. the disease cannot spread within the nursery, Scenario 1) and *R*_0_ = 5 (i.e. the disease spreads fast within the nursery, Scenario 2). Although the value of *R*_0_ will depend on the characteristics of the particular disease and the plant, given that established human diseases can have values up to the mid teens (measles has a value of *R*_0_ = 12–18) and that many human diseases have basic reproductive numbers in the realms of 5 (Anderson and May, 1991), values of *R*_0_ have rarely been found in plants diseases. Even though, one study has found that *R*_0_ is of the order of 50 for wheat stripe rust in large wheat fields (Mikaberidze et al., 2014). Moreover, the values of *R*_0_ is a factor that depends not only on disease traits, but also on the properties of the nursery. For example, actions like the routine application of fungicides, the routine cleaning of equipment or the arranging the nursery to limit contact between plants could lower *R*_0_. Consequently, one could consider Scenario 1 as the case where the nursery has effective cleanliness whereas Scenario 2 is where there is a lack of effective cleanliness.

For the loss of revenue from selling an infected plant, we consider a value of *L* = 10 as our baseline, which implies that the costs of removal are the same as the losses made from selling an infected plant; this would be compared to scenarios with smaller values for *L*, in particular, in Scenario 1b, *L* = 5 and in Scenario 2b, *L* = 1. It is reasonable to assume that smaller values of *L* would correspond to situations where the diseased plants have superficial damage and/or there are secondary markets for infected plant outputs with little difference in the net price of healthy mature plants. Higher values of *L* correspond to diseases that have a large impact on the net price of a highly valuable plant, without an effective secondary market for infected plants. In particular, plants with that take a long time to mature or bespoke plants sold to the landscape sector tend to sell for higher prices and thus prone to large losses from infection.

Lastly, for the cost-effectiveness parameter, we consider *d* = 1 as the baseline. *d* = 1 corresponds with a (1 − exp ^−1^) × 100%(≈63%) reduction in the proportion of infected plants coming into the nursery (*p*(*u*_*ins*_)) with an additional unit in restriction (solid red line in Fig. 2). For comparison, we assume *d* = 0.3 for scenarios where the disease is costly to restrict (Scenario 1c and 2c). Using *d* = 0.3 corresponds with a (1 − exp ^−0.3^) × 100%(≈26%) reduction in *p*(*u*_*ins*_) when the restriction costs increase by one unit (solid blue line in Fig. 2). Traits of systems where *d* is large are where it is easy to detect infected plant inputs, because either the inputs have symptoms that can be spotted by eye or there exist diagnostic technology that is cheap, quick and easy to use. On the other hand, traits of systems where *d* is small are measures that require a lot of labour, time or machinery to detect infected plant inputs. We suspect that this is often true for bacteria, viruses and such with no clear symptoms in infected inputs, which need expensive and potentially time-consuming tests to detect infected inputs.

Putting this all together, we have six different cases, three of which are where the disease is not particular infectious (which will collectively be known as Scenario 1) and three of which consider a highly infectious disease (collectively known as Scenario 2). A summary of all six Scenarios, including results, is in Table 1.

## 3 Results

### 3.1 Long term disease dynamics

#### 3.1.1 Perfect restriction (*p*(*u*_*ins*_) = 0)

**Fig. 3:**
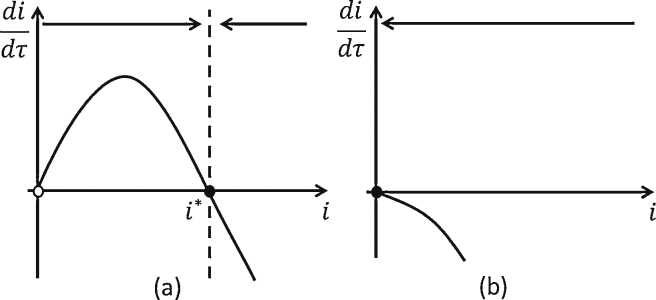
Perfect restriction (*p* = 0). (a) If $R_{0}^{\mathrm{rem}}=\frac{R_{0}}{1+u_{\mathrm{rem}}}>1$, then the prevalence equation is negative for all positive prevalence. There is one non-negative steady state, i^*^ = 0, which is stable. then the prevalence equation is a form of logistic growth. There are two steady states (where $\frac{\mathrm{di}}{d\tau }$), i^*^ = 0 and $i^{*}=1-\frac{1}{R_{0}^{\mathrm{rem}}}$. *i* = 0 is unstable and that for the region between *i* = 0 and $i=1-\frac{1}{R_{0}^{\mathrm{rem}}}$, $\frac{\mathrm{di}}{d\tau }>0$ and thus disease prevalence will increase over time (represented by the arrow at the top). (b) If $R_{0}^{\mathrm{rem}}<1$, then the prevalence equation is negative for all positive prevalence. There is one non-negative steady state, i^*^ = 0, which is stable. Note that when *u*_*rem*_ = 0, $R_{0}^{\mathrm{rem}}=R_{0}$.

In the absence of the removal of infected plants (i.e. *u*_*rem*_ = 0), we have two cases: (1) *R*_0_ < 1: In this case, on average, a single infected plant infects less than one susceptible plant over the lifetime of the infected plant and hence the disease will die out eventually. Consequently, the only stable state is the disease-free state and thus the disease cannot become endemic (i^*^ = 0) (Fig. 3(b)). (2) *R*_0_ > 1: Here, a single infected plant infects more than one susceptible over the lifetime of the infection and hence the disease will spread out from any single introduction. Hence, the only stable steady state is the endemic steady state $i^{*}=1-\frac{1}{R_{0}}$ and thus any introduction will result in the disease being endemic (Fig. 3(a)).

In the presence of the removal of infected plants (i.e. *u*_*rem*_ > 0), the results are similar to the absence of removal, except the threshold between a disease-free nursery and an endemic disease in the nursery is based on value of $R_{0}^{\mathrm{rem}}=\frac{R_{0}}{1+u_{\mathrm{rem}}}$. For $R_{0}^{\mathrm{rem}}>1$, for any introduction of disease, the disease will invade and approach the steady state $i^{*}=1-\frac{1}{R_{0}^{\mathrm{rem}}}$ (Fig. 3(a)). For $R_{0}^{\mathrm{rem}}<1$, the disease will not become endemic from any single introduction (Fig. 3(b)).

Now, for *u*_*rem*_ > 0, we have that $R_{0}^{\mathrm{rem}}<R_{0}$. Thus, the disease will find it harder to survive as infected plants have less time in the nursery to infect other plants because of removal. In particular, if the removal effort (*u*_*rem*_) is sufficiently large (*u*_*rem*_ > *R*_0_ − 1), we can reduce $R_{0}^{\mathrm{rem}}$ below 1 and consequently rid the nursery of the disease in the long run.

#### 3.1.2 Imperfect restriction (*p*(*u*_*ins*_) = *p* > 0)

**Fig. 4:**
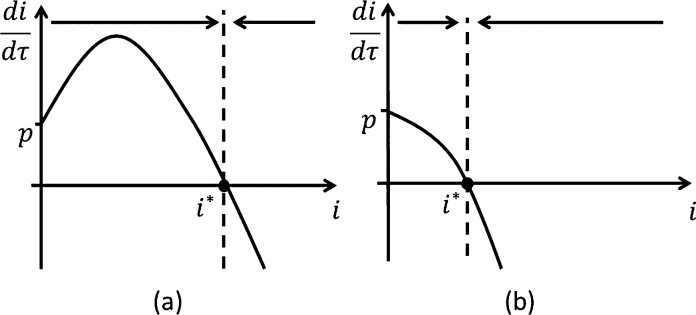
Imperfect restriction (*p* > 0). (a) $R_{0}^{p}=\frac{R_{0}}{1+u_{\mathrm{rem}}(1-p)}>1$ and (b) $R_{0}^{p}=\frac{R_{0}}{1+u_{\mathrm{rem}}(1-p)}<1$. For both figures have only one steady state that is stable; there is no disease-free steady state unlike the case with *p* = 0.

With imperfect restriction, the disease will always persist in the nursery plant stock to some level (Fig. 4). There is always only one steady state that is non-negative,

(11) $$i^{*}=\frac{R_{0}-1-(1-p)u_{\mathrm{rem}}+\sqrt{{\left(R_{0}-1-(1-p)u_{\mathrm{rem}}\right)}^{2}+4pR_{0}}}{2R_{0}},$$

and it is always stable. The lack of a disease-free steady state is due to the constant inflow of infected plants into the system. In particular, $\frac{\mathrm{di}}{d\tau }=p>0$ at *i* = 0 and thus disease prevalence will always increase when starting with a disease-free nursery.

Despite the disease always persisting in the nursery, we wish to distinguish between two cases. If $R_{0}^{p}=\frac{R_{0}}{1+u_{\mathrm{rem}}(1-p)}>1$ (Fig. 4(a)), the disease spreads through the plant stock like before. Notice that $R_{0}>R_{0}^{p}>R_{0}^{\mathrm{rem}}$. This is because the removal control is only effective (1 − *p*) × 100% of the time, since *p* × 100% of the time in the removing infected is replaced by another infected. In particular, if *p* = 0, $R_{0}^{p}=R_{0}^{\mathrm{rem}}$, whereas for *p* = 1, $R_{0}^{p}=R_{0}$. Consequently, imperfect restriction undermines the removal control. In particular, if $R_{0}^{\mathrm{rem}}>1$, the disease would persist without any infected inputs (as shown in the previous subsection for perfect restriction). If $\frac{R_{0}}{1+u_{\mathrm{rem}}(1-p)}<1$ (Fig. 4(b)); the disease does not spread effectively within the nursery and instead its persistence in the nursery is dependent on constant introduction of infected plant inputs into the nursery.

The disease dynamics for the imperfect restriction are essentially logistic growth with an additional constant introduction of infected plants. In particular, Fig. 4(a) can be seen as a shifted and transformed version of the logistic growth in Fig. 3(a), which results in the loss of the disease-free steady state and an increase in the endemic steady state. Likewise, Fig. 4(b) can be seen as a shifted version of the ‘negative logistic growth’ in Fig. 3(b), where the disease-free steady state becomes an endemic steady state.

Table 2 summarises the results about when the disease is endemic in the nursery for both the perfect and imperfect restriction.

### 3.2 Optimal management: Analytical results

Working with the prevalence steady state, we seek to find the optimal combination of removal and restriction, *u*_*rem*_ and *u*_*ins*_ that minimises the costs of the plant disease at the nursery:

(12) $$Q=(L+{\mathrm{Cu}}_{\mathrm{rem}})i^{*}+u_{\mathrm{ins}}=(L+{\mathrm{Cu}}_{\mathrm{rem}})\frac{M+\sqrt{M^{2}+4R_{0}p(u_{\mathrm{ins}})}}{2R_{0}}+u_{\mathrm{ins}}$$

where *M*(*u*_*ins*_, *u*_*rem*_) = *R*_0_ − 1 − (1 − *p*(*u*_*ins*_))*u*_*rem*_. Note, *M* is fundamentally linked with $R_{0}^{p}$ with equivalent threshold properties: *M* = 0 corresponds with $R_{0}^{p}=1$, *M* > 0 corresponds with $R_{0}^{p}>1$ and *M* < 0 corresponds with $R_{0}^{p}<1$.

To find the combination of *u*_*rem*_ and *u*_*ins*_ that minimise *Q*, we need to consider the partial derivatives of *Q* to find internal and boundary minima. When optimal prevention and control policies are interior they satisfy the first order conditions:

(13) $$\frac{\partial Q}{\partial u_{\mathrm{rem}}}={\mathrm{MC}}_{\mathrm{rem}}-{\mathrm{MB}}_{\mathrm{rem}}=0$$

(14) $$\frac{\partial Q}{\partial u_{\mathrm{ins}}}={\mathrm{MC}}_{\mathrm{ins}}-{\mathrm{MB}}_{\mathrm{ins}}=0$$

where

$$\begin{array}{cc} {\mathrm{MB}}_{\mathrm{rem}} & =\frac{\left(L+{\mathrm{Cu}}_{\mathrm{rem}})(1-p(u_{\mathrm{ins}})\right)}{2R_{0}}\left(1+\frac{M}{\sqrt{M^{2}+4R_{0}p(u_{\mathrm{ins}})}}\right)\\ {\mathrm{MC}}_{\mathrm{rem}} & =\frac{C}{2R_{0}}\left(M+\sqrt{M^{2}+4R_{0}p(u_{\mathrm{ins}})}\right)\\ {\mathrm{MB}}_{\mathrm{ins}} & =-\frac{(L+{\mathrm{Cu}}_{\mathrm{rem}})\frac{\partial p(u_{\mathrm{ins}})}{\partial u_{\mathrm{ins}}}}{2R_{0}}\left(u_{\mathrm{rem}}+\frac{{\mathrm{Mu}}_{\mathrm{rem}}+2R_{0}}{\sqrt{M^{2}+4R_{0}p(u_{\mathrm{ins}})}}\right)\\ {\mathrm{MC}}_{\mathrm{ins}} & =1 \end{array}$$

As expected, Eq. (13) (Eq. (14)) requires a nursery owner to allocate resources to removal (restriction) until the last dollar spent on removal (restriction) equals the marginal benefits gained in terms of reduction in infection costs. The analysis of the properties of local and global minima for removal (Eq. (13)) and restriction (Eq. (14)), can be found in Appendix A, Appendix A, respectively.

Looking at Eqs. (13), (14) and incorporating the results found in Appendix A, Appendix A, we have the following:

• With respect to removal, if MB_*rem*_>MC_*rem*_ at *u*_*rem*_ = 0 then MB_*rem*_>MC_*rem*_ for all *u*_*rem*_ and thus *u*_*rem*_ = *u*_*remmax*_ is the global minimum with respect to *u*_*rem*_.
• If MB_*rem*_<MC_*rem*_ at *u*_*rem*_ = *u*_*remmax*_ then MB_*rem*_<MC_*rem*_ for all admissible *u*_*rem*_ and thus *u*_*rem*_ = 0, i.e. no removal effort, is the global minimum with respect to *u*_*rem*_.
• The only other case with respect to *u*_*rem*_ is that there exists a value of *u*_*rem*_ ∈ (0, *u*_*remmax*_) such that MB_*rem*_ =MC_*rem*_, and this internal solution is a local maximum. Both *u*_*rem*_ = 0 and *u*_*rem*_ = *u*_*remmax*_ are local minima with respect to *u*_*rem*_. One of these will be the global minimum with respect to *u*_*rem*_ and direct comparison of the values of *Q* at these local minima is required.
• With respect to restriction, if MB_*ins*_<MC_*ins*_ at *u*_*ins*_ = 0, then MB_*ins*_<MC_*ins*_ for all *u*_*ins*_ > 0 and thus *Q* is minimised at *u*_*ins*_ = 0, i.e. no restriction is optimal.
• Conversely, if MB_*ins*_>MC_*ins*_ for *u*_*ins*_ = 0 (for fixed *u*_*rem*_), then there is a value of *u*_*ins*_ > 0 such that MB_*ins*_ =MC_*ins*_ (i.e. a level of restriction where the marginal benefit is equal to the marginal cost), and this value is the global minimum with respect to *u*_*ins*_, i.e. moderate restriction is optimal.
• One can analyse whether removal and restriction work together as complements or as substitutes by analysing $\frac{\partial^{2}Q}{\partial u_{\mathrm{ins}}\partial u_{\mathrm{rem}}}$. For complements, $\frac{\partial^{2}Q}{\partial u_{\mathrm{ins}}\partial u_{\mathrm{rem}}}<0$ (since *Q* represents costs, not profit or utility) and $\frac{\partial^{2}Q}{\partial u_{\mathrm{ins}}\partial u_{\mathrm{rem}}}>0$ for substitutes. The expression for $\frac{\partial^{2}Q}{\partial u_{\mathrm{ins}}\partial u_{\mathrm{rem}}}$ is complex and can be either sign. In particular, if *M* and *R*_0_ are large and *u*_*rem*_ is zero, then $\frac{\partial^{2}Q}{\partial u_{\mathrm{ins}}\partial u_{\mathrm{rem}}}<0$ and thus restriction and removal are complements; whereas, if *u*_*rem*_ is large and thus *M* is large and negative, $\frac{\partial^{2}Q}{\partial u_{\mathrm{ins}}\partial u_{\mathrm{rem}}}>0$, making restriction and removal substitutes.

From this and by looking at Eqs. (13) and (14), we can establish some rules of thumb. Firstly, by looking at Eq. (14), we can see that increasing *L* and/or *C*, will increase the marginal benefits in damages avoided and thus generally results in higher restriction (in particular, it never leads to lower levels of restriction). Secondly, looking at Eq. (13), we can see that increasing *L* and *C* proportionally results in no change in whether *u*_*rem*_ = 0 or *u*_*rem*_ = *u*_*remmax*_ are optimal. Consequently, the values of *L* and *C* themselves have no impact on the optimal strategy for removal, only the ratio between *L* and *C* (in other words, the nursery owner would apply the same effort if losses for an infected plant were $1 and removal costs $1 as $10 losses with $10 removal costs, it is just a matter of scale). This is not the case for *u*_*ins*_, since both, revenue losses and removal costs are compared with the cost of restriction.

The effects of *R*_0_ and the parameters in *p*(*u*_*ins*_) on Eqs. (13), (14) are not straightforward, partly because they are also included within *M*, although the presence of $\frac{\partial p(u_{\mathrm{ins}})}{\partial u_{\mathrm{ins}}}$ in *MB*_*ins*_ suggests that increasing the cost-effectiveness of restriction, *d*, increases *MB*_*ins*_ around *u*_*ins*_ = 0, making restriction measures more likely.

### 3.3 Optimal management: numerical solutions

Table 1 provides a summary of the results for all the scenarios analysed.

#### 3.3.1 Scenario 1: low infectiousness

Scenario 1 represents cases of diseases that would not persist in the nursery without the constant introduction of infected plant materials. First we will consider the baseline case where *L* = 10 and *d* = 1 (Scenario 1a), before focusing on the effects a reduction in *L* (to *L* = 5) has on the optimal solution (Scenario 1b) and then consider the effect of reducing the effectiveness per dollar in restriction effort *d* to 0.3 (Scenario 1c).

**Fig. 5:**
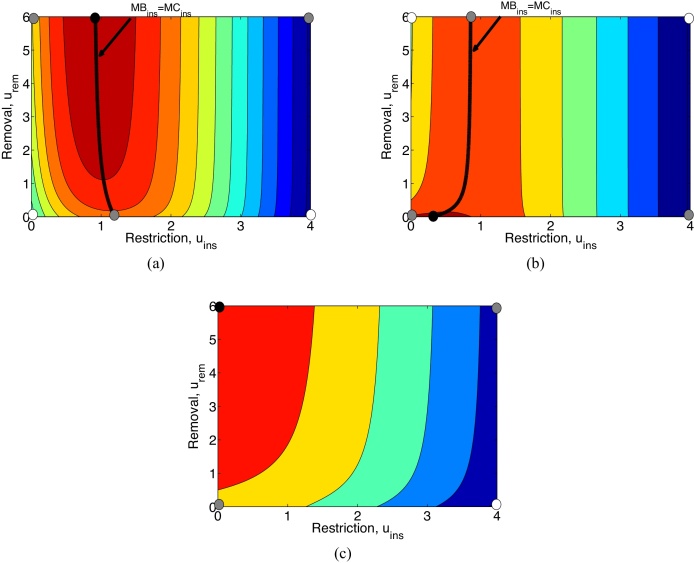
Contour plots of *Q* with respect to both removal and restriction for (a) Scenario 1a, (b) Scenario 1b and (c) Scenario 1c. Red regions are the regions of lowest costs whereas blue regions signify highest costs. The black solid line represents MB_*ins*_=MC_*ins*_ (there are no lines for removal in this Scenario). Black dots are local minima, white dots are local maxima and grey dots are saddle points (points on the right boundary are local maxima/saddle point if we limit *u*_*ins*_ to regions in these figures). *R*_0_, *L* and *d* are given in Table 1. Other parameters: *C* = 10, *a* = 0.2 and *b* = 0. (For interpretation of the references to colour in this figure, the reader is referred to the web version of this article.)

In Scenario 1a (Fig. 5(a)), we have that the marginal benefit of removal is always greater than the marginal cost $\left(\mathrm{since}\;\frac{\partial Q}{\partial u_{\mathrm{rem}}}<0\;\mathrm{at}\;u_{\mathrm{rem}}=0\right)$. Consequently, the optimal removal is maximum removal *u*_*rem*_ = *u*_*remmax*_. This is to be expected, since removing an infected plant prevents not only losses from that infected plants (which are assumed to be equal to the removal cost, *L* = *C*) but also losses from secondary infections. Given that *R*_0_ > *p*(*u*_*ins*_) this additional loss from secondary infections is considerably greater than the potential loss that could result from the possibility of buying infected inputs when replacing plants that were subject to removal.

In Fig. 5(a) and all other contour plots, the optimal level of restriction is determined by the line MB_*ins*_=MC_*ins*_. For Scenario 1a (Fig. 5(a)), with no removal effort, the optimal level of restriction is around *u*_*ins*_ = 1.2. As the nursery increases its capacity to remove infected plants, it slowly reduces the optimal level of restriction.

Next, we consider the case where the revenue losses from infection are considerably lower (Scenario 1b, Fig. 5(b)). Reducing the revenue losses from infection from *L* = 10 to *L* = 5 has made removal less viable. It is better to leave an infected plant in the nursery, because the costs of removing and replacing an infected plant is too expensive relative to the revenue loss associated to its lower net price.

Now, in contrast to Scenario 1a, Scenario 1c (Fig. 5(c)) simulates a situation where restriction is more costly. This is represented by decreasing *d* from 1 to 0.3 and consequently spending an extra unit in restriction results in a reduction in infected inputs of (1 − exp ^−0.3^) * 100%(≈26%), considerably worse than the 63% in Scenario 1a. This decrease in *d* has shifted the optimal restriction line where MB_*ins*_=MC_*ins*_ to the left, in this case the line is now to the left of the *y*-axis and thus beyond the realms of reality, and consequently restriction has become inviable. Thus the optimal strategy in Scenario 1c is maximum removal with no restriction (Fig. 5(c)).

#### 3.3.2 Scenario 2: high infectiousness

Increasing the basic reproduction number from *R*_0_ = 0.5 (Scenario 1) to *R*_0_ = 5 (Scenario 2) increases the complexity of the results.

**Fig. 6:**
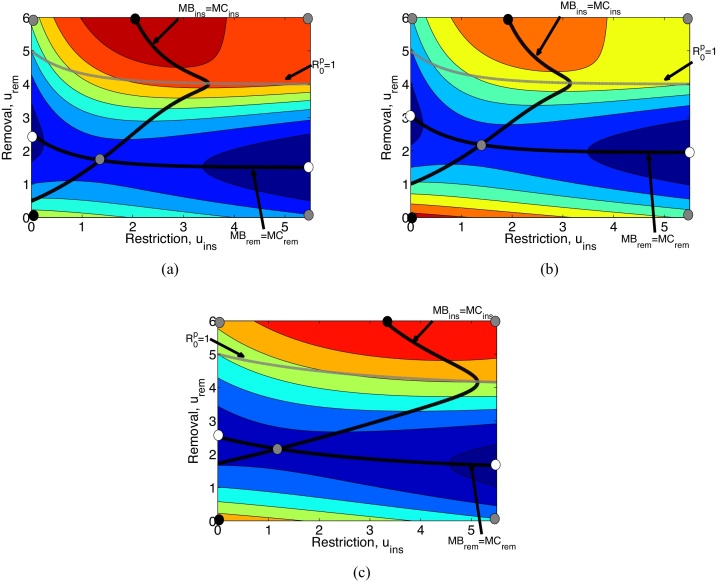
Contour plots of profit *Q* with respect to both removal and restriction for (a) Scenario 2a, (b) Scenario 2b and (c) Scenario 2c. Red regions are the regions of lowest costs whereas blue regions signify highest costs. The black lines represent MB_*ins*_=MC_*ins*_ and MB_*rem*_=MC_*rem*_ whereas the grey line represents the values of (*u*_*ins*_, *u*_*rem*_) that correspond to $R_{0}^{p}=1$. The dots have the same meaning as Fig. 5(a). *R*_0_, *L* and *d* are given in Table 1. Other parameters are the same as Fig. 5. (For interpretation of the references to colour in this figure, the reader is referred to the web version of this article.)

When a disease is highly infectious, any small introduction of infected plants will spread the disease through the nursery quickly. Consequently, investing in restriction does not prevent the disease going through the plants growing in the nursery. However, restriction does have a mild effect on disease prevalence when prevalence in the nursery is high as the ‘cleaner’ inputted plants that replace those leaving the nursery will have a mild rinsing effect. Thus, without removal effort, restriction is often not viable (i.e. no restriction is optimal) when the disease is highly infectious. This is particularly the case here when contrasting the viable restriction in Scenario 1a (Fig. 5(a) where *R*_0_ = 0.5) and the inviable restriction in Scenario 2a (Fig. 6(a)) when there is no removal.

In Scenario 2a (Fig. 6(a)) there are up to two local minima. We know from the analytical results that optimal removal is either *u*_*rem*_ = 0 or *u*_*rem*_ = *u*_*remmax*_. Consequently we can argue about the importance of *u*_*remmax*_ by varying *u*_*rem*_ = *u*_*remmax*_ in the contour plots, following the MB_*ins*_=MC_*ins*_ line. If the nursery capacity to remove is small, in particular such that *u*_*remmax*_ is below the intersection of the MB_*ins*_=MC_*ins*_ and MB_*rem*_=MC_*rem*_ curves, then there is only one local (and thus global) minimum, which is to do nothing and let the disease take its course. If *u*_*remmax*_ is beyond the intersection, then there are two local minima, the aforementioned ‘do nothing’ and *u*_*rem*_ = *u*_*remmax*_ with the corresponding restriction level given by MB_*ins*_=MC_*ins*_. The global minimum is one of these two local minima and which one depends on the value of *u*_*remmax*_; if *u*_*remmax*_ is small enough that the contour is either blue or green (below *u*_*remmax*_ ≈ 3.5) then ‘do nothing’ is optimal, whereas beyond *u*_*remmax*_ ≈ 3.5 where the contours are yellow to red, then maximum removal (*u*_*rem*_ = *u*_*remmax*_) is the optimal strategy. Consequently, there is a great range of values *u*_*remmax*_ where the optimal solution is to ‘do nothing’, that it is futile to try and control the disease without being able to really get on top of it.

One particularly interesting result in Scenario 2a (Fig. 6(a)) is the kink that occurs in the MB_*ins*_=MC_*ins*_ curve. This kink occurs indistinguishably close to $R_{0}^{p}=1$ since the kink occurs around where the MB_*ins*_=MC_*ins*_ and $R_{0}^{p}=1$ curves intersect. Below this kink, we have that increasing level of removal is linked with increasing level of restriction, i.e. removal and restrictions are complements. This occurs since restriction improves the effectiveness of removal as it reduces the chances that an infected plant, which has been removed, is replaced by another infected plant. However, above the kink, we have that increasing level of removal results in a decrease in the optimal level of restriction, i.e. they are substitutes. This agrees with the final bullet point of the analytical results, where restriction and removal are complements when *R*_0_ is large and *u*_*rem*_ is small, whereas restriction and removal are substitutes when *u*_*rem*_ is substantially larger than *R*_0_.

Going from Scenario 2a to 2b (Fig. 6(b)), there is a reduction in the loss in revenue from selling an infected plant from *L* = 10 to *L* = 1 (note that this is a considerably smaller revenue loss than in Scenario 1b). The effect of this small revenue loss in the optimal effort of controlling the disease is relatively minor with respect to Scenario 2a; MB_*ins*_=MC_*ins*_ has shifted a little to the left, and thus the optimal level of restriction is reduced everywhere and MB_*rem*_=MC_*rem*_ has shifted a bit to the right and a little up. The consequence of the move in MB_*rem*_=MC_*rem*_ is that removal is also less viable everywhere. In particular, the intersection between these two lines that separates the two local minima has shifted up, increasing the region where there is only one local minimum; and consequently, ‘do nothing’ has become the optimal control irrespective to the value of *u*_*remmax*_.

Notice that *L* has to be really small to achieve the result above. For *L* = 5, the global minimum is maximum removal as long as *u*_*remmax*_ is sufficiently above the kink around $R_{0}^{p}=1$ (figure not given, use Fig. 6(a) as guide). Conversely, a large increase in revenue losses, *L*, is needed to exclude ‘do nothing’ as a local optimal minimum; first, optimal restriction expenditure becomes positive for zero removal around *L* = 25 (i.e. MB_*ins*_=MC_*ins*_ intercepts the *x*-axis), and this ‘restriction only state’ becomes a local minimum. The ‘restriction only state’ remains a local minimum while the curves representing MB_*ins*_=MC_*ins*_ and MB_*rem*_=MC_*rem*_ intercept. This intercept disappears around *L* = 45, beyond which there is no ‘zero-removal’ local minimum. This means that even for large revenue losses, if the nursery capacity to remove is small (*u*_*remmax*_ small) then the nursery is very likely to be in the region where no expenditure in removal is optimal. This is because the disease will still spread through the nursery since $R_{0}^{p}$ is still considerably larger than 1, making removal efforts futile.

Now, consider the case where restriction is less cost-effective as *d* is decreased to 0.3 (Scenario 2c, Fig. 6(c)). This decrease has a relatively minor effect on the removal line MB_*rem*_=MC_*rem*_ in Fig. 6(c), the line keeps the same intercept with the *y*-axis and it is flatter than in Fig. 6(a). This is predictable since decreasing cost-effectiveness means that more needs to be spent in restriction in order to have the same effect in the reduction of the probability of buying infected inputs. Likewise, the line of MB_*ins*_=MC_*ins*_ has (a) a higher intercept with the *y*-axis, making restriction less worthy if there is low removal, and (b) at the kink the expenditure on restriction has increased. The latter effect is due to the reduction in the cost-effectiveness (essentially an increase in the price of a 50% reduction in infected inputs) which does reduce restriction effort, but it does increase total spending on restriction.

## 4 Discussion and conclusions

In this paper, we have analysed the prevention and control management options available to a nursery owner in order to minimise the impacts of an infectious disease that may spread within the nursery. To this end, we derived a bioeconomic model of a plant nursery, where the manager can opt either to restrict the proportion of infected plant material coming into the nursery (prevention), or remove infected plants within the nursery (control), or a combination of both strategies. We assume that there is an upper limit on removal effort. Our analytical results show that (a) if infected inputs are always coming into the nursery, the disease would persist in the nursery, and will approach a unique endemic steady state (Section 3.1.2 and Fig. 4); (b) the optimal removal is either maximum removal (i.e. the upper limit in removal efforts given the nursery's capacity) or no removal, as long as restriction efforts are optimally allocated, i.e. where the marginal cost of restriction equals its marginal benefit in terms of disease damages avoided (Section 3.2); (c) optimal restriction expenditure increase with both the revenue losses for selling mature infected plants and costs of removal; while maximal removal is more likely to be optimal if either revenue infection losses increase or removal costs decrease (Section 3.2); (d) since any removed infected plant stock needs to be replaced buying new plant inputs, which could potentially be infected, the manager can increase the effectiveness of removal effort by increasing restriction effort (see expressions of $R_{0}^{p}$ and i^*^ in Section 3.1.2).

The numerical analysis of the Scenarios (summarised in Table 1) with varying conditions in the level of infectiousness of the disease, damages to the nursery, and cost-effectiveness of management efforts, highlights three relevant results for private biosecurity decisions. First, results indicate that it is optimal to spend on maximum removal efforts unless the revenue losses from selling infected mature plants are considerable lower than the cost of removal (especially for highly infectious diseases, e.g. Scenario 2).

Secondly, if the capacity to remove infected plants is very limited, due for example to temporal or monetary constrains, it may be optimal to ‘do nothing’ (again, particularly for highly infectious diseases, Scenario 2). It is only worth removing infected plants if the efforts applied can limit the expansion of the disease through secondary infections within the nursery, otherwise removal resources could be waste; it is not worthwhile removing an infected plant if the replaced plant will likely become infected. The private benefits of removal efforts in curbing the disease have therefore threshold properties. Benefits can only be achieved once at least a minimum amount has been contributed to their production. This property on removal efforts is expected to affect the probability of cooperating (e.g. Sandler, 2004, Touza and Perrings, 2011), when strategic decisions among private agents is relevant to limit the probability of outbreaks (e.g. Hennessy, 2008, Epanchin-Niell and Wilen, 2015).

A third result is the finding of synergies between restriction and removal strategies, which are determined by the reproduction number, i.e. how contagious a disease is and could be spread through trade. This contributes to previous existing literature that only focus on substitutionary effects between prevention and control. For example, Olson and Roy (2005) examine the conditions under which the optimal policy relies solely on either prevention or control. Kim et al. (2006) examine the optimal combination of pre-discovery prevention, post-discovery prevention and post-discovery control where the discovery time is stochastic, and find that post-discovery prevention and control are substitutes. Leung et al. (2005) consider that if there is expensive control activities, this reduces social welfare at the post-invasion state, and consequently higher social welfare can be achieved from avoiding invasion, and substituting control by prevention efforts. Similarly, Finnoff et al. (2007) conclude that a risk averse agent would substitute more prevention expenditures with control policies when compared to a risk neutral agent. Here, we found that the optimal level of restriction is complementary with removal efforts if the disease is beyond the nursery owner's ability to limit its spread. The underlying reason for this is that, restriction measures may not be very effective in the case of highly infectious diseases (Scenario 2), since some infected plants materials will always get past the restriction regime, and once infected plants are in the nursery the disease will spread fast within the nursery. In those situations, if the manager increases the level of effort in removing infected plants, the disease becomes more manageable, and consequently making expenditures in restriction measures more effective. In addition, increased efforts on restriction makes also removal more effective, reducing the probability of buying infected inputs when the nursery owner has to buy new stock to replace those infected plants that were removed. Consequently, removal and restriction efforts are complementary for highly infected diseases.

This phenomenon where ‘prevention’ and ‘cure’ are complementary has been found in the human health literature in Hey and Patel (1983) and Hennessy (2008). Hennessy (2008) argue that for ‘prevention’ and ‘cure’ being complements is that increasing prevention reduces the chance that cured individuals become sick again and thus improving the long term benefit of curing sick individuals. This argument is analogous to the reasons that can explain why restriction improves the effectiveness of removal in Scenario 2, as the replacement of a removed infected plant with an infected plant can be seen as (instantaneous) reinfection.

We also show that this complementary relationship between prevention and control continues as removal level increase until around $R_{0}^{p}=1$. Beyond this point the disease no longer is able to spread through the nursery and instead relies on the constant introduction of infected plant inputs to persist in the nursery. In this case, the disease could be manageable through the removal programme, and the nursery owner can choose whether to remove it once it is in the nursery or prevent it from entering the nursery. This means, restriction and removal efforts are substitutes, akin to the classic ‘prevention vs cure’ argument.

However, it should be noted that the analysis in this paper is based on the long term dynamics of the disease and decision making, thus our work fits more the endemic stage of an infection with the nursery being subject to continual invasion pressure. Consequently, it neglects the epidemic/invasion stage, and uncertain benefits from delaying the spread of the disease through prevention and/or surveillance during this stage (e.g. Haight and Polasky, 2010, Mehta et al., 2007). Moreover, we also recognise that many nurseries work on a shorter term basis than used in this model. For example, some nurseries are seasonal and only have a generation or two of plants in the nursery for one season before an annual reset of the nursery, with new plants stock. In this case, a steady state might not be appropriate analysis as not enough time has occurred for a steady state to be reached. Following the above literature, in cases like those in Scenario 2 with highly infectious diseases, restriction and removal may be more viable in the early stages of disease introduction (unlike the long term) since they can delay the inevitable disease spreading through the nursery. However, even in shorter time-scales, equilibrium-based analysis form a strong baseline for understanding optimal decisions.

In the model derivation process we assumed that the nursery stock is fixed (i.e. the nursery is always full). This is not always true, especially if seasonal effects (like weather or seasonal demand) occur or if the nursery owner reduces the size of the nursery as a disease management tool. During periods with a reduced nursery stock, the basic reproductive number *R*_0_ is reduced (since the disease is density dependent) as is the cost-effectiveness of restriction, ‘*d*’. The reduction in *R*_0_ means the disease will spread less within the nursery and thus is easier to control by removal. Consequently, the constant full nursery assumption used in this paper gives an upper limit to the extent of the disease will spread and thus a worst case scenario in terms of uncontrolled damages from a pathogen. On top of that, the reduction on *R*_0_ from a lower *N* reduces the range of *u*_*rem*_ where restriction and removal are complements. On the other hand, the reduction in the cost-effectiveness of restriction would result in a less stringent restriction regime (i.e. an increase in the proportion of infected plant inputs, *p*(*u*_*ins*_)), akin to what is found when comparing Scenarios 1a and 2a with Scenarios 1c and 2c.

In this paper, we have assumed the disease is an SI disease, i.e. each plant is either susceptible or infected and there is no recovery from the disease. This was for simplicity and generality. However, many plant diseases have recovery, latency, asymptomatic infection and immunity, as well as free-living stages in the environment (i.e. in the soil or water). The presence of asymptomatic and latent infected plant inputs undermines the owner's ability to restrict infected inputs coming into the nursery since identifying infected plants material inputs becomes much more complex or even impossible if no symptoms of infection or clear evidence of pathogens are present. In addition, our analysis only focuses on diseases that can only enter the nursery via infected plant material inputs (i.e. though plant trade). However, for many different nurseries, pathogens and pests get into the nursery through a number of different pathways. In particular, contaminated water is often the reason for *Phytophthora* and other pathogens getting into plant nurseries (Hong and Moorman, 2005, and references therein). We suspect that in this situation, restriction strategies that focus on inspecting plant inputs would have a limited effect on preventing the diseases, which would reduce their cost-effectiveness and therefore their optimal level of provision.

The level of restriction in this paper depends greatly on the choice of the function *p*(*u*_*ins*_), the proportion of infected plant material inputs that are infected for a given level of restriction. In this paper, we used an exponentially decreasing function to obtain numerical results since it was the simplest function that satisfies the desired properties of *p*(*u*_*ins*_) (i.e. which, in short, is monotonic decreasing of *u*_*ins*_). This function has the property that the first dollar spent on restriction is always the most effective, and that each dollar spent has a smaller effect on *p*(*u*_*ins*_) than the previous dollar. This property would not necessarily be appropriate in several cases. For example, functions where a small investment in restriction has little effect and a substantial investment that more has to spent for a restriction regime to start to have a noticeable effect on the proportion of infected plant materials coming in could be more appropriate if substantial funds are needed for effective levels of knowledge, labour, machinery and skills to be maintained. A suggested simple function that could provide useful incite into management satisfies this property is $(a-b)\exp^{-du_{\mathrm{ins}}^{2}}+b$ (in which case the most cost-effective level of restriction is at *u*_*ins*_ = (2*d*)^−1/2^).

Finally, note that this paper deals with one disease of concern for the nursery owner to control. Generally, a nursery owner has a multitude of diseases to be concerned about. For example, the tomato *Solanum lycopersicum* is known to be a host for over 500 different pests and pathogens (CABI, 2015). Likewise, a nursery can have many pathogens present. For example, at least 13 different species of *Phytophthora* were found in the irrigation water at three nurseries in northern Germany in 1995 (Themann et al., 2002, Brasier, 2008). Likewise, in Bavaria in 2002, there were five different species of *Phytophthora* found in the soil around a single open-planted alder seedling (T. Jung, LWF, D-85354 Freising, personal communication cited in Brasier, 2008). With a multitude of diseases to manage, a common optimal strategy on restriction and removal would be needed, a strategy that would likely differ from the strategy of each of the diseases in isolation.

## Figures and Tables

**Table 1:** The Scenarios and their key results.

Scenario	*R*_0_	*L*	*d*	↓*p*	Optimal result
1a	0.5	10	1	63%	Maximum removal with restriction
1b	0.5	5	1	63%	No removal with restriction
1c	0.5	10	0.3	26%	Maximum removal, no restriction
2a	5	10	1	63%	‘Do nothing’ if *u*_*remmax*_ ≲ 3.5, else maximum removal with restriction
2b	5	1	1	63%	‘Do nothing’ is optimal everywhere
2c	5	10	0.3	26%	‘Do nothing’ if *u*_*remmax*_ ≲ 4.75, else maximum removal with restriction

Here, ‘↓*p*’ is the reduction of infected inputs from an increase in costs of restriction in one unit (i.e. (1− exp(−*d*)) × 100 % rounded to the nearest percentage point). ‘Do nothing’ means zero removal and zero restriction.

**Table 2:** Summary of constant control.

	Endemic	Disease-free
Perfect restriction, no removal	*R*_0_ > 1	*R*_0_ < 1
Perfect restriction with removal	$R_{0}^{\mathrm{rem}}>1$	$R_{0}^{\mathrm{rem}}<1$
Imperfect restriction	Always	Never

Here, $R_{0}^{\mathrm{rem}}=\frac{R_{0}}{1+u_{\mathrm{rem}}}$.

## Appendix A Optimal solution with respect to *u*_*rem*_: ‘all or nothing’

To find out what the optimal solutions with respect to *u*_*rem*_, we need to investigate:

(A.1) $$\frac{\partial Q}{\partial u_{\mathrm{rem}}}=(L+{\mathrm{Cu}}_{\mathrm{rem}})\frac{\frac{\partial M}{\partial u_{\mathrm{rem}}}+\frac{2M\frac{\partial M}{\partial u_{\mathrm{rem}}}}{2\sqrt{M^{2}+4R_{0}p(u_{\mathrm{ins}})}}}{2R_{0}}+C\frac{M+\sqrt{M^{2}+4R_{0}p(u_{\mathrm{ins}})}}{2R_{0}}=0,$$

where *M*(*u*_*ins*_, *u*_*rem*_) = *R*_0_ − 1 − (1 − *p*(*u*_*ins*_))*u*_*rem*_. First, we need to manipulate this into something more manageable.

(A.2) $$\begin{array}{cc} \frac{\partial Q}{\partial u_{\mathrm{rem}}}= & -\frac{\left(L+{\mathrm{Cu}}_{\mathrm{rem}})(1-p(u_{\mathrm{ins}})\right)}{2R_{0}}\left(1+\frac{M}{\sqrt{M^{2}+4R_{0}p(u_{\mathrm{ins}})}}\right)+\frac{C}{2R_{0}}\left(M+\sqrt{M^{2}+4R_{0}p(u_{\mathrm{ins}})}\right) \end{array}$$

(A.3) $$\begin{array}{cc} = & -\frac{C}{2R_{0}}\left(\left(\frac{L}{C}+u_{\mathrm{rem}}\right)(1-p(u_{\mathrm{ins}}))\left(1+\frac{M}{\sqrt{M^{2}+4R_{0}p(u_{\mathrm{ins}})}}\right)-\left(M+\sqrt{M^{2}+4R_{0}p(u_{\mathrm{ins}})}\right)\right) \end{array}$$

(A.4) $$\begin{array}{cc} = & -\frac{C}{2R_{0}\sqrt{M^{2}+4R_{0}p(u_{\mathrm{ins}})}}\left(\left(\left(\frac{L}{C}+u_{\mathrm{rem}}\right)(1-p(u_{\mathrm{ins}}))-M\right)\left(\sqrt{M^{2}+4R_{0}p(u_{\mathrm{ins}})}+M\right)-4R_{0}p(u_{\mathrm{ins}})\right) \end{array}$$

Consequently, solutions of $\frac{\partial Q}{\partial u_{\mathrm{rem}}}=0$ are solutions of $\left(\left(\frac{L}{C}+u_{\mathrm{rem}}\right)(1-p(u_{\mathrm{ins}}))-M\right)(\sqrt{M^{2}+4R_{0}p(u_{\mathrm{ins}})}+M)-4R_{0}p(u_{\mathrm{ins}})=0$. Now, if such solutions exist and are admissible, we need to find out if one of these solution is a maximum with respect *u*_*rem*_. To do so, we need to look at the second derivative.

(A.5) $$\begin{array}{cc} \frac{\partial^{2}Q}{\partial u_{\mathrm{rem}}^{2}}= & -\frac{C}{2R_{0}}\frac{\partial M}{\partial u_{\mathrm{ins}}}\frac{\partial }{\partial M}\left(\frac{1}{\sqrt{M^{2}+4R_{0}p(u_{\mathrm{ins}})}}\right)\overset{=0}{\overset{︷}{\left(\left(\left(\frac{L}{C}+u_{\mathrm{rem}}\right)(1-p(u_{\mathrm{ins}}))-M\right)\left(\sqrt{M^{2}+4R_{0}p(u_{\mathrm{ins}})}+M\right)-4R_{0}p(u_{\mathrm{ins}})\right)}}-\frac{C}{2R_{0}\sqrt{M^{2}+4R_{0}p(u_{\mathrm{ins}})}}\left((1-p(u_{\mathrm{ins}}))\left(\sqrt{M^{2}+4R_{0}p(u_{\mathrm{ins}})}+M\right)-\frac{\partial M}{\partial u_{\mathrm{ins}}}\left(1+\frac{M}{\sqrt{M^{2}+4R_{0}p(u_{\mathrm{ins}})}}\right)\right)\left(+\left(\frac{L}{C}+u_{\mathrm{rem}}\right)(1-p(u_{\mathrm{ins}}))\frac{\partial M}{\partial u_{\mathrm{ins}}}\frac{\partial }{\partial M}\left(\sqrt{M^{2}+4R_{0}p(u_{\mathrm{ins}})}\right)\right) \end{array}$$

(A.6) $$\begin{array}{cc} = & -\frac{C}{2R_{0}\sqrt{M^{2}+4R_{0}p(u_{\mathrm{ins}})}}\left(2(1-p(u_{\mathrm{ins}}))\left(\sqrt{M^{2}+4R_{0}p(u_{\mathrm{ins}})}+M\right)\right)-\left((1-p(u_{\mathrm{ins}}))\left(\left(\frac{L}{C}+u_{\mathrm{rem}}\right)(1-p(u_{\mathrm{ins}}))-M\right)\left(1+\frac{M}{\sqrt{M^{2}+4R_{0}p(u_{\mathrm{ins}})}}\right)\right) \end{array}$$

(A.7) $$\begin{array}{cc} = & -\frac{C(1-p(u_{\mathrm{ins}}))}{2R_{0}(M^{2}+4R_{0}p(u_{\mathrm{ins}}))}\left(2\left(M^{2}+4R_{0}p(u_{\mathrm{ins}})+M\sqrt{M^{2}+4R_{0}p(u_{\mathrm{ins}})}\right)\right)-\left(\left(\left(\frac{L}{C}+u_{\mathrm{rem}}\right)(1-p(u_{\mathrm{ins}}))-M\right)\left(\sqrt{M^{2}+4R_{0}p(u_{\mathrm{ins}})}+M\right)\right) \end{array}$$

(A.8) $$\begin{array}{cc} = & -\frac{C(1-p(u_{\mathrm{ins}}))}{2R_{0}(M^{2}+4R_{0}p(u_{\mathrm{ins}}))}\left(2\left(M^{2}+2R_{0}p(u_{\mathrm{ins}})+M\sqrt{M^{2}+4R_{0}p(u_{\mathrm{ins}})}\right)\right)-\left(\overset{=0}{\overset{︷}{\left(\left(\left(\left(\frac{L}{C}+u_{\mathrm{rem}}\right)(1-p(u_{\mathrm{ins}}))-M\right)\left(\sqrt{M^{2}+4R_{0}p(u_{\mathrm{ins}})}+M\right)\right)-4R_{0}p(u_{\mathrm{ins}})\right)}}\right) \end{array}$$

(A.9) $$=-\frac{C(1-p(u_{\mathrm{ins}}))}{R_{0}(M^{2}+4R_{0}p(u_{\mathrm{ins}}))}\left(M^{2}+2R_{0}p(u_{\mathrm{ins}})+M\sqrt{M^{2}+4R_{0}p(u_{\mathrm{ins}})}\right)$$

If *M* > 0, then $\frac{\partial^{2}Q}{\partial u_{\mathrm{rem}}^{2}}<0$ and thus all internal solutions are local maxima with respect to *u*_*rem*_. It is not completely clear if this is the case for *M* < 0 so instead look to find the value of *M* where $M^{2}+2R_{0}p(u_{\mathrm{ins}})+M\sqrt{M^{2}+4R_{0}p(u_{\mathrm{ins}})}$ has its minimum. So we look at the properties of solutions of $\frac{\partial }{\partial M}\left(M^{2}+2R_{0}p(u_{\mathrm{ins}})+M\sqrt{M^{2}+4R_{0}p(u_{\mathrm{ins}})}\right)=0$.

(A.10) $$\begin{array}{cc} \frac{\partial }{\partial M}\left(M^{2}+2R_{0}p(u_{\mathrm{ins}})+M\sqrt{M^{2}+4R_{0}p(u_{\mathrm{ins}})}\right)= & 2M+\sqrt{M^{2}+4R_{0}p(u_{\mathrm{ins}})}+\frac{M^{2}}{\sqrt{M^{2}+4R_{0}p(u_{\mathrm{ins}})}} \end{array}$$

(A.11) $$\begin{array}{cc} = & \frac{2}{\sqrt{M^{2}+4R_{0}p(u_{\mathrm{ins}})}}\left(M^{2}+2R_{0}p(u_{\mathrm{ins}})+M\sqrt{M^{2}+4R_{0}p(u_{\mathrm{ins}})}\right)=0 \end{array}$$

Solutions of this satisfy $M=-\frac{M^{2}+2R_{0}p(u_{\mathrm{ins}})}{\sqrt{M^{2}+4R_{0}p(u_{\mathrm{ins}})}}$. Substituting this into $M^{2}+2R_{0}p(u_{\mathrm{ins}})+M\sqrt{M^{2}+4R_{0}p(u_{\mathrm{ins}})}$ gives:

(A.12) $$\begin{array}{cc} & -\frac{M^{2}+2R_{0}p(u_{\mathrm{ins}})}{\sqrt{M^{2}+4R_{0}p(u_{\mathrm{ins}})}}\left(-\frac{M^{2}+2R_{0}p(u_{\mathrm{ins}})}{\sqrt{M^{2}+4R_{0}p(u_{\mathrm{ins}})}}+\sqrt{M^{2}+4R_{0}p(u_{\mathrm{ins}})}\right)+2R_{0}p(u_{\mathrm{ins}})\\ & =-\frac{M^{2}+2R_{0}p(u_{\mathrm{ins}})}{M^{2}+4R_{0}p(u_{\mathrm{ins}})}\left(-(M^{2}+2R_{0}p(u_{\mathrm{ins}}))+M^{2}+4R_{0}p(u_{\mathrm{ins}})\right)+2R_{0}p(u_{\mathrm{ins}})\\ & =2R_{0}p(u_{\mathrm{ins}})\left(1-\frac{M^{2}+2R_{0}p(u_{\mathrm{ins}})}{M^{2}+4R_{0}p(u_{\mathrm{ins}})}\right)>0 \end{array}$$

and thus $M^{2}+2R_{0}p(u_{\mathrm{ins}})+M\sqrt{M^{2}+4R_{0}p(u_{\mathrm{ins}})}>0$ always and thus $\frac{\partial^{2}Q}{\partial u_{\mathrm{rem}}^{2}}>0$ and thus internal solutions are always local maxima with respect to *u*_*rem*_. As there is no internal minimum with respect to *u*_*rem*_, the global minimum must occur on the boundary, either at *u*_*rem*_ = 0 or *u*_*rem*_ = *u*_*remmax*_. If $\frac{\partial Q}{\partial u_{\mathrm{rem}}}<0$ at *u*_*rem*_ = 0 then *u*_*rem*_ = 0 is a local (global) maximum and *u*_*rem*_ = *u*_*remmax*_ is the global minimum. Conversely, if $\frac{\partial Q}{\partial u_{\mathrm{rem}}}>0$ at *u*_*rem*_ = *u*_*remmax*_ then *u*_*rem*_ = *u*_*remmax*_ is a local (global) maximum and thus *u*_*rem*_ = 0 is a global minimum. If $\frac{\partial Q}{\partial u_{\mathrm{rem}}}>0$ at *u*_*rem*_ = 0 and $\frac{\partial Q}{\partial u_{\mathrm{rem}}}<0$ at *u*_*rem*_ = *u*_*remmax*_, then you have must compare *Q* for *u*_*rem*_ = 0 and *u*_*rem*_ = *u*_*remmax*_ since both are local minima.

## Appendix B Optimal control with respect to restriction *u*_*ins*_: ‘do something or do nothing’

We need to find out the global minimum with respect to restriction *u*_*ins*_ by analysing:

(B.1) $$\frac{\partial Q}{\partial u_{\mathrm{ins}}}=(L+{\mathrm{Cu}}_{\mathrm{rem}})\frac{\frac{\partial M}{\partial u_{\mathrm{ins}}}+\frac{2M\frac{\partial M}{\partial u_{\mathrm{ins}}}+4R_{0}\frac{\partial p(u_{\mathrm{ins}})}{\partial u_{\mathrm{ins}}}}{2\sqrt{M^{2}+4R_{0}p(u_{\mathrm{ins}})}}}{2R_{0}}+1=0.$$

where *M*(*u*_*ins*_, *u*_*rem*_) = *R*_0_ − 1 − (1 − *p*(*u*_*ins*_))*u*_*rem*_. First, we will look at the second partial derivative to see if $\frac{\partial Q}{\partial u_{\mathrm{ins}}}$ is an increasing or decreasing function of *u*_*ins*_:

(B.2) $$\begin{array}{cc} \frac{\partial^{2}Q}{\partial u_{\mathrm{ins}}^{2}}= & \frac{\partial^{2}p(u_{\mathrm{ins}})}{\partial u_{\mathrm{ins}}^{2}}\frac{\left(L+{\mathrm{Cu}}_{\mathrm{rem}}\right)}{2R_{0}}\left(u_{\mathrm{rem}}+\frac{{\mathrm{Mu}}_{\mathrm{rem}}+2R_{0}}{\sqrt{M^{2}+4R_{0}p(u_{\mathrm{ins}})}}\right)+{\left(\frac{\partial p(u_{\mathrm{ins}})}{\partial u_{\mathrm{ins}}}\right)}^{2}\frac{\left(L+{\mathrm{Cu}}_{\mathrm{rem}}\right)}{2R_{0}}\left(\frac{-2R_{0}({\mathrm{Mu}}_{\mathrm{rem}}+2R_{0})}{{\left(M^{2}+4R_{0}p(u_{\mathrm{ins}})\right)}^{\frac{3}{2}}}\right) \end{array}$$

(B.3) $$\begin{array}{cc} = & \frac{\partial^{2}p(u_{\mathrm{ins}})}{\partial u_{\mathrm{ins}}^{2}}\frac{L+{\mathrm{Cu}}_{\mathrm{rem}}}{2R_{0}}\left(u_{\mathrm{rem}}+\frac{\left({\mathrm{Mu}}_{\mathrm{rem}}+2R_{0})(M^{2}+4R_{0}p(u_{\mathrm{ins}}))-2R_{0}\frac{{\left(\frac{\partial p}{\partial u_{\mathrm{ins}}}\right)}^{2}}{\frac{\partial^{2}p}{\partial u_{\mathrm{ins}}^{2}}}({\mathrm{Mu}}_{\mathrm{rem}}+2R_{0}\right)}{{\left(M^{2}+4R_{0}p(u_{\mathrm{ins}})\right)}^{\frac{3}{2}}}\right) \end{array}$$

(B.4) $$\begin{array}{cc} = & \frac{\partial^{2}p(u_{\mathrm{ins}})}{\partial u_{\mathrm{ins}}^{2}}\frac{L+{\mathrm{Cu}}_{\mathrm{rem}}}{2R_{0}}\left(u_{\mathrm{rem}}+\frac{M^{2}+4R_{0}p(u_{\mathrm{ins}})-2R_{0}\frac{{\left(\frac{\partial p}{\partial u_{\mathrm{ins}}}\right)}^{2}}{\frac{\partial^{2}p}{\partial u_{\mathrm{ins}}^{2}}}}{M^{2}+4R_{0}p(u_{\mathrm{ins}})}\underset{\mathrm{always}\;>-u_{\mathrm{rem}}}{\underset{︸}{\frac{{\mathrm{Mu}}_{\mathrm{rem}}+2R_{0}}{\sqrt{M^{2}+4R_{0}p(u_{\mathrm{ins}})}}}}\right) \end{array}$$

Now, since we do not have sufficient knowledge on the properties of $\frac{\partial^{2}p}{\partial u_{\mathrm{ins}}^{2}}$ in general, we will continue with *p*(*u*_*ins*_) = *b* + (*a* − *b*) exp(−*du*_*ins*_). Thus $\frac{\partial p}{\partial u_{\mathrm{ins}}}=-d(a-b)\exp(-{\mathrm{du}}_{\mathrm{ins}})=-d(p(u_{\mathrm{ins}})-b)$ and $\frac{\partial^{2}p}{\partial u_{\mathrm{ins}}^{2}}=-d\frac{\partial p}{\partial u_{\mathrm{ins}}}=d^{2}(a-b)\exp(-{\mathrm{du}}_{\mathrm{ins}})=d^{2}(p(u_{\mathrm{ins}})-b)$. Armed with this, we have:

(B.5) $$\begin{array}{cc} \frac{\partial^{2}Q}{\partial u_{\mathrm{ins}}^{2}} & =\frac{\left(L+{\mathrm{Cu}}_{\mathrm{rem}})d^{2}(p(u_{\mathrm{ins}})-b\right)}{2R_{0}}\left(\underset{>0}{\underset{︸}{u_{\mathrm{rem}}+\overset{\in (0,1)}{\overset{︷}{\frac{M^{2}+2R_{0}(p(u_{\mathrm{ins}})+b)}{M^{2}+4R_{0}p(u_{\mathrm{ins}})}}}{\overset{︷}{\frac{{\mathrm{Mu}}_{\mathrm{rem}}+2R_{0}}{\sqrt{M^{2}+4R_{0}p(u_{\mathrm{ins}})}}}}^{\mathrm{always}\;>-u_{\mathrm{rem}}}}}\right) \end{array}$$

(B.6) $$\begin{array}{cc} & >0\;\mathrm{when}\;L+{\mathrm{Cu}}_{\mathrm{rem}}>0 \end{array}$$

Firstly, we note that if *L* + *Cu*_*rem*_ ≤ 0 (which could be true if *L* < 0), there are no internal solutions from possible for Eq. (14) from the main text and we have $\frac{\partial Q}{\partial u_{\mathrm{ins}}}=0$ is monotonically increasing to −1. Hence, $\frac{\partial Q}{\partial u_{\mathrm{ins}}}<0$ always and thus zero restriction is always the best (a disease that is beneficial should not be restricted). For *L* + *Cu*_*rem*_ > 0, we have that $\frac{\partial Q}{\partial u_{\mathrm{ins}}}$ is monotonically increasing (to 1 as *u*_*ins*_→ ∞). In other words, increasing restriction has even diminishing returns, reducing the marginal benefit, whereas the marginal cost remains the same. Given we have that $\frac{\partial Q}{\partial u_{\mathrm{ins}}}$ is monotonically increasing to 1 (and is continuous), we know that there exists one and only one admissible solution with respect to *u*_*ins*_ (for fixed *u*_*rem*_) if $\frac{\partial Q}{\partial u_{\mathrm{ins}}}<0$ at *u*_*ins*_ = 0 and that this solution is a global minimum with respect to *u*_*ins*_, i.e. the optimal control involves some restriction. Otherwise, $\frac{\partial Q}{\partial u_{\mathrm{ins}}}\geq 0$ at *u*_*ins*_ = 0, there is no internal solution and the global minimum with respect to *u*_*ins*_ is at *u*_*ins*_ = 0, i.e. no restriction is optimal.

If such solutions do not exist within admissible controls (*u*_*rem*_ ∈ [0, *u*_*remmax*_] and *u*_*ins*_ ≥ 0), we need to pick the minimising values on the boundary, i.e. if $\frac{\partial Q}{\partial u_{\mathrm{ins}}}>0$ at *u*_*ins*_ = 0, then either *u*_*ins*_ = 0 and *u*_*ins*_ =∞ are the global maximum. However, since $\frac{\partial Q}{\partial u_{\mathrm{ins}}}\to 1$ as *u*_*ins*_→ ∞ (because *p*(*u*_*ins*_) is converging to *b* and thus $\frac{\partial p(u_{\mathrm{ins}})}{\partial u_{\mathrm{ins}}}\to 0$, *u*_*ins*_ =∞ is always a local maximum and thus *u*_*ins*_ = 0 is the global minimum, i.e. the cost minimising strategy, when $\frac{\partial Q}{\partial u_{\mathrm{ins}}}>0$ at *u*_*ins*_ = 0.

## Appendix C Linking dynamic and stationary approaches

Taking Eq. (6) and following the rescaling and rearrangement that occur between Eq. (7) and (9) leads to:

(C.1) $$\min_{u_{\mathrm{ins}},u_{\mathrm{rem}}}\int_{0}^{\hat{T}}e^{-\hat{r}t}\left((L+{\mathrm{Cu}}_{\mathrm{rem}})i+u_{\mathrm{ins}}\right)\mathrm{dt}$$

where $\hat{T}=T\delta$ and $\hat{r}=\frac{r}{\delta }$ (henceforth, we will drop these hats for simplicity, being consistent with what was done in the main text). First, we establish and analyse the Hamiltonian of Eqs. (9), (10). This Hamiltonian is:

(C.2) $$H=e^{-\mathrm{rt}}\left((-L-{\mathrm{Cu}}_{\mathrm{rem}})i-u_{\mathrm{ins}}\right)+\lambda \left(p(u_{\mathrm{ins}})(1+u_{\mathrm{rem}}i)-i-u_{\mathrm{rem}}i+R_{0}(1-i)i\right).$$

Consequently, the adjoint equation is:

(C.3) $$\frac{d\lambda }{\mathrm{dt}}=-\frac{\partial H}{\partial i}=-(e^{-\mathrm{rt}}(-L-{\mathrm{Cu}}_{\mathrm{rem}})+\lambda (p(u_{\mathrm{ins}})u_{\mathrm{rem}}-1-u_{\mathrm{rem}}+R_{0}(1-2i))).$$

The optimality conditions for *u*_*ins*_ and *u*_*rem*_ are:

(C.4) $$\frac{\partial H}{\partial u_{\mathrm{ins}}}=-e^{-\mathrm{rt}}+\lambda \left(\frac{\partial p(u_{\mathrm{ins}})}{\partial u_{\mathrm{ins}}}(1+u_{\mathrm{rem}}i)\right)=0$$

and

(C.5) $$\frac{\partial H}{\partial u_{\mathrm{rem}}}=i(\lambda (p(u_{\mathrm{ins}})-1)-{\mathrm{Ce}}^{-\mathrm{rt}})=0,$$

respectively.

To link the solutions in this paper to those of this Hamiltonian, we will assume an infinite time interval, and treat *u*_*rem*_, *u*_*ins*_ as constants. On top of this, we will insert the steady state value of *i*^*^ from Eq. (11) given from the population dynamics. Rearranging (C.4) gives:

(C.6) $$\lambda =\frac{e^{-\mathrm{rt}}}{\left(\frac{\partial p(u_{\mathrm{ins}})}{\partial u_{\mathrm{ins}}}(1+u_{\mathrm{rem}}i)\right)}.$$

Inserting this into (C.3) gives:

(C.7) $$\frac{d\lambda }{\mathrm{dt}}=e^{-\mathrm{rt}}\left(L+{\mathrm{Cu}}_{\mathrm{rem}}+\frac{p(u_{\mathrm{ins}})u_{\mathrm{rem}}-1-u_{\mathrm{rem}}+R_{0}(1-2i)}{\frac{\partial p(u_{\mathrm{ins}})}{\partial u_{\mathrm{ins}}}(1+u_{\mathrm{rem}}i)}\right).$$

From this, using the constant *u*_*rem*_, *u*_*ins*_ and *i*^*^ assumption and assuming *λ* = 0 at infinity, gives:

(C.8) $$\lambda =-\frac{1}{r}e^{-\mathrm{rt}}\left(L+{\mathrm{Cu}}_{\mathrm{rem}}+\frac{p(u_{\mathrm{ins}})u_{\mathrm{rem}}-1-u_{\mathrm{rem}}+R_{0}(1-2i)}{\frac{\partial p(u_{\mathrm{ins}})}{\partial u_{\mathrm{ins}}}(1+u_{\mathrm{rem}}i)}\right).$$

Using the two expressions for *λ* (C.6), (C.8), we get:

(C.9) $$\frac{1}{\frac{\partial p(u_{\mathrm{ins}})}{\partial u_{\mathrm{ins}}}(1+u_{\mathrm{rem}}i)}=-\frac{1}{r}\left(L+{\mathrm{Cu}}_{\mathrm{rem}}+\frac{p(u_{\mathrm{ins}})u_{\mathrm{rem}}-1-u_{\mathrm{rem}}+R_{0}(1-2i)}{\frac{\partial p(u_{\mathrm{ins}})}{\partial u_{\mathrm{ins}}}(1+u_{\mathrm{rem}}i)}\right).$$

Inserting $i^{*}=\frac{M+\sqrt{M^{2}+4p(u_{\mathrm{ins}})R_{0}}}{2R_{0}}$, where *M* = *R*_0_ − 1 − (1 − *p*(*u*_*ins*_))*u*_*rem*_, and with a little rearranging, we arrive at:

(C.10) $$r=-\left((L+{\mathrm{Cu}}_{\mathrm{rem}})\frac{\partial p(u_{\mathrm{ins}})}{\partial u_{\mathrm{ins}}}\left(1+u_{\mathrm{rem}}\frac{M+\sqrt{M^{2}+4pR_{0}}}{2R_{0}}\right)+\sqrt{M^{2}+4pR_{0}}\right).$$

Dividing everything by $-\sqrt{M^{2}+4p(u_{\mathrm{ins}})R_{0}}$ and rearranging gives:

(C.11) $$-\frac{r}{\sqrt{M^{2}+4p(u_{\mathrm{ins}})R_{0}}}=1-\left(-\frac{(L+{\mathrm{Cu}}_{\mathrm{rem}})\frac{\partial p(u_{\mathrm{ins}})}{\partial u_{\mathrm{ins}}}}{2R_{0}}\left(u_{\mathrm{rem}}+\frac{{\mathrm{Mu}}_{\mathrm{rem}}+2R_{0}}{\sqrt{M^{2}+4R_{0}p(u_{\mathrm{ins}})}}\right)\right).$$

Notice that the right hand side is $\frac{\mathrm{dQ}}{{\mathrm{du}}_{\mathrm{ins}}}={\mathrm{MC}}_{\mathrm{ins}}-{\mathrm{MB}}_{\mathrm{ins}}$ from Eq. (14). Thus for zero discounting (*r* = 0), $\frac{\mathrm{dQ}}{{\mathrm{du}}_{\mathrm{ins}}}=0$ gives the optimal restriction, whereas for a positive discounting rate (*r* > 0), the optimal restriction satisfies $\frac{\mathrm{dQ}}{{\mathrm{du}}_{\mathrm{ins}}}=-\frac{r}{\sqrt{M^{2}+4R_{0}p(u_{\mathrm{ins}})}}$. However, since $\frac{\mathrm{dQ}}{{\mathrm{du}}_{\mathrm{ins}}}$ is monotonically increasing function, we know that increasing the discount rate (*r*) would lower the optimal level of restriction. This effect is very dependent on how long the plant is expected to be in the nursery due to the time rescaling (i.e. since $\hat{r}=\frac{r}{\delta }$). If the average plant stay is short (i.e. weeks to months) then this discounting effect is negligible, whereas for longer period (i.e. years), this term becomes larger, having more impact on the optimal restriction.

Moving on to optimal removal, (C.5) is generally never satisfied, and instead the optimal removal is a ‘bang–bang’ control (i.e. all or nothing) which is consistent with the static analysis. Consequently, the optimal solution is either *u*_*rem*_ = 0 or *u*_*rem*_ = *u*_*remmax*_, which depends on the sign of *λ*(*p*(*u*_*ins*_) − 1) − *Ce*^−*rt*^.

To determine the sign, we will focus on the threshold *λ*(*p*(*u*_*ins*_) − 1) − *Ce*^−*rt*^ = 0. Substituting Eq. (C.6) and rearranging gives:

(C.12) $$C\frac{\partial p(u_{\mathrm{ins}})}{\partial u_{\mathrm{ins}}}(1+u_{\mathrm{rem}}i)=-(1-p(u_{\mathrm{ins}})).$$

Now, rearranging Eq. (C.9) and inserting the steady state value of *i*^*^ from Eq. (11) gives

(C.13) $$\frac{\partial p(u_{\mathrm{ins}})}{\partial u_{\mathrm{ins}}}(1+u_{\mathrm{rem}}i)=-\frac{r+\sqrt{M^{2}+4p(u_{\mathrm{ins}})R_{0}}}{L+{\mathrm{Cu}}_{\mathrm{rem}}}.$$

Substituting this into (C.12) and arranging gives

(C.14) $$\mathrm{Cr}=(L+{\mathrm{Cu}}_{\mathrm{rem}})(1-p(u_{\mathrm{ins}}))-C\sqrt{M^{2}+4p(u_{\mathrm{ins}})R_{0}}.$$

Multiplying by $\frac{-1}{2R_{0}}\left(1+\frac{M}{\sqrt{M^{2}+4R_{0}p(u_{\mathrm{ins}})}}\right)$ we arrive at:

(C.15) $$\begin{array}{c} -\frac{\mathrm{rC}}{2R_{0}}\left(1+\frac{M}{\sqrt{M^{2}+4R_{0}p(u_{\mathrm{ins}})}}\right)=\frac{C}{2R_{0}}(M+\sqrt{M^{2}+4p(u_{\mathrm{ins}})R_{0}})-\frac{\left(L+{\mathrm{Cu}}_{\mathrm{rem}})(1-p(u_{\mathrm{ins}})\right)}{2R_{0}}\left(1+\frac{M}{\sqrt{M^{2}+4R_{0}p(u_{\mathrm{ins}})}}\right). \end{array}$$

This of condition is analogous with the static problem, with the right hand side being $\frac{\mathrm{dQ}}{{\mathrm{du}}_{\mathrm{rem}}}={\mathrm{MC}}_{\mathrm{rem}}-{\mathrm{MB}}_{\mathrm{rem}}$ from Eq. (13).

This alone does not give the global optimal since there are two *λ*'s to compare, one where *u*_*rem*_ = 0, the other where *u*_*rem*_ = *u*_*remmax*_. In cases where *λ*(*u*_*rem*_ = 0)(*p*(*u*_*ins*_) − 1) − *Ce*^−*rt*^ < 0 but *λ*(*u*_*rem*_ = *u*_*remmax*_)(*p*(*u*_*ins*_) − 1) − *Ce*^−*rt*^ > 0, a comparison in terms of profit must be made, which is analogous to the two local optima solutions found in the static solutions. Again, like with restriction, we have that no discounting gives the same result, and increasing the discount rate makes *u*_*rem*_ = *u*_*remmax*_ less likely to be globally optimal.

## Footnotes

1 Although there is no recovery, infected plants can leave the system via being sold on or being removed and be replaced by a susceptible plant. This means there is some kind of pseudo-recovery, meaning the system behaves more like a classic SIS system than SI.

2 Another approach is to have assume that infected plants stay longer in the nursery due to slower growth. However, this approach would ultimately lead to the same reduction in revenue, since revenue is price × output. Consequently, the only real difference would be that different output rates would lead to a more complex replacement term.

3 We assume a fixed price for plant outputs and inputs for simplicity. However, it has been suggested that nurseries work under monopolistic competition (Barbier et al., 2011).

4 A few diseases can be beneficial, e.g. mild infestations of *Botrytis cinerea* on grapes results in noble rot, which is desirable for dessert wines; in such cases where *P*_*I*_ > *P*_*S*_, the optimal control is always to do nothing, which is trivial.
